# Enhanced Expression of Plasminogen Activators and Inhibitor in the Healing of Tympanic Membrane Perforation in Rats

**DOI:** 10.1007/s10162-023-00891-5

**Published:** 2023-02-21

**Authors:** Maria Makuszewska, Magdalena Cieślińska, Maria M. Winnicka, Bożena Skotnicka, Kazimierz Niemczyk, Tomasz Bonda

**Affiliations:** 1 Department of Otorhinolaryngology, Head and Neck Surgery, Medical University of Warsaw, Banacha 1a, 02-097 Warsaw, Poland; 2 Department of General and Experimental Pathology, Medical University of Białystok, Mickiewicza 2c, 15-222 Białystok, Poland; 3 Department of Pediatric Otolaryngology, Medical University of Białystok, Waszyngtona 17, 15-274 Białystok, Poland

**Keywords:** Tympanic membrane perforation, Tissue-type plasminogen activator, Urokinase-type plasminogen activator, Urokinase-type plasminogen activator receptor, Plasminogen activator inhibitor type 1, Rats

## Abstract

The significance of plasminogen activation during the tympanic membrane (TM) healing is known mainly from studies performed on knock-out mice. In the previous study, we reported activation of genes coding proteins of plasminogen activation and inhibition system in rat’s TM perforation healing. The aim of the present study was the evaluation of protein products expressed by these genes and their tissue distribution using Western blotting and immunofluorescent method, respectively, during 10-day observation period after injury. Otomicroscopical and histological evaluation were employed to assess the healing process. The expression of urokinase plasminogen activator (uPA) and its receptor (uPAR) were significantly upregulated in the proliferation phase, with subsequent gradual attenuation during remodeling phase of healing process, when keratinocyte migration was weakening. The expression of plasminogen activator inhibitor type 1 (PAI-1) also showed the highest levels during the proliferation phase. The increase of tissue plasminogen activator (tPA) expression was observed during the whole observation period, with the highest activity during the remodeling phase. Immunofluorescence of these proteins was present mainly in migrating epithelium. Our study found that plasminogen activation (uPA, uPAR, tPA) and inhibitory (PAI-1) molecules form a well-structured regulatory system of the epithelial migration that is critical to the healing of TM after its perforation.

**Supplementary Information:**

The online version contains supplementary material available at 10.1007/s10162-023-00891-5.

## Introduction

**Fig. 1:**
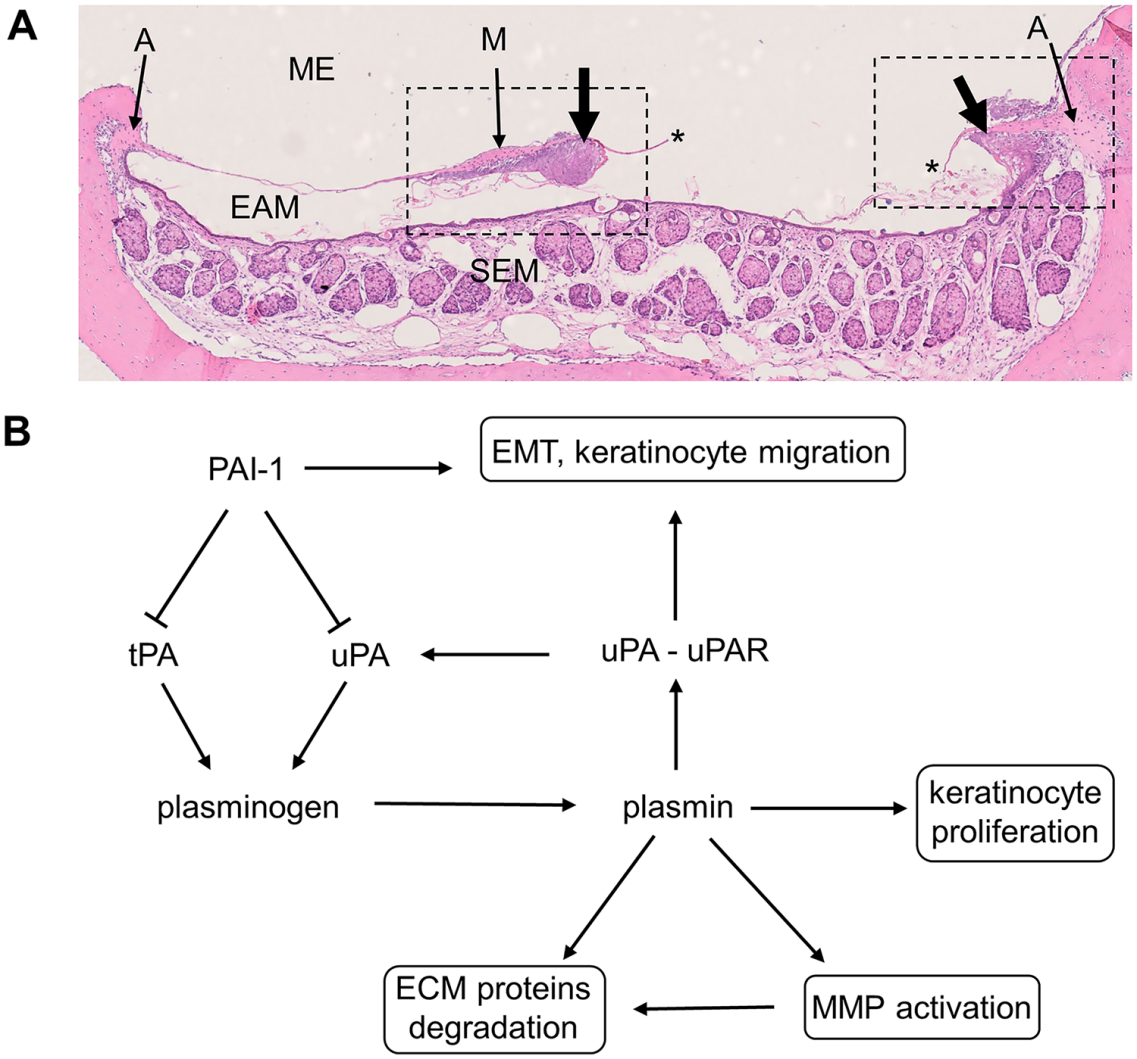
**A** Horizontal section through pars tensa of healing TM on day 3 after injury. ME, middle ear; EAM, external acoustic meatus; SEM, skin of external acoustic meatus; A, tympanic annulus; M, malleus handle (near umbo); single asterisk indicates edge of the perforation; thick arrows, proliferating squamous epithelium near malleus handle and annulus on the side of the perforation. Rectangles marked with a dashed line demarcate the regions of TM presented on Fig. 2 (right rectangle) and Fig. 3 (left rectangle). **B** The organization of the plasminogen activation/inhibition system and its influence on processes involved in epidermal healing, which are presented within frames. ECM, extracellular matrix; EMT, epithelial to mesenchymal transition; MMP, metalloproteinases

The tympanic membrane (TM) is the structure suspended in the air between the middle ear cavity and the external meatus. For this reason, the course of its healing has to be different than in injured skin, as it does not start from the bottom of the wound with granulation tissue formation but from the edges of perforation [1–7]. The healing of TM injury, after short hemostasis and inflammatory phases, starts with the proliferation and migration of the keratinizing squamous epithelium from the regions of the malleus handle and annulus, towards the perforation. The regions of malleus handle and annulus are called proliferative zones as they contain progenitor or stem cells [4, 8, 9]. The proliferating and migrating epithelium create the heaped mass over the edges of perforation and gradually covers it (Fig. 1A). Over the perforation, migrating epithelial cells seem to be guided by the crust and keratin spur. Epithelial to mesenchymal transition (EMT), that is essential for accomplishing this process, requires potent pericellular proteolysis which is executed principally by plasminogen activation system. The involvement of particular molecules of plasminogen system in EMT initiation and progression was proven in experiments performed on cell lines. Moreover, it has been shown that EMT activation required both PAI-1 expression and uPAR signaling [10–12]. The proliferation of the connective tissue occurs secondarily, in the direct proximity of the proliferating and migrating epithelium [3–7].

Although there is no typical fibrin- and fibronectin-rich, provisional matrix formation, tissue remodeling plays crucial role in TM healing, especially in proliferation and remodeling phases. Two major proteolytic systems, plasminogen activator system and the matrix metalloproteinases, seem to be involved in this process [13–15].

Plasminogen is produced by liver and circulates in the blood [16]. The concentration of plasminogen in the body fluids is supposed to be at a steady-state level, and the formation of plasmin is assumed to be regulated by the accessibility and activity of the plasminogen activation and inhibition system. Accumulation of plasminogen takes place largely in wounds, and its extent directly correlates with circulating plasminogen concentrations [17]. The transport of plasminogen to wounds is predominantly accomplished by its binding to plasminogen receptors present on inflammatory cells, mainly macrophages and granulocytes [17, 18]. Moreover, it has been shown that plasminogen is synthesized also in normal human differentiated epidermal keratinocytes of the stratum granulosum, where it is largely associated with corneocyte envelopes and is not serum-born [19].

Plasmin is the key molecule of plasminogen activator system. It is generated from its precursor—plasminogen—by tissue or urokinase-type plasminogen activators. Plasminogen activation is also regulated by the presence of cell surface receptors for plasminogen activators and specific inhibitors [20]. A scheme illustrating the involvement of these proteins in healing process is presented in Fig. 1B. It has been shown that in plasminogen-deficient mice, skin wound healing is delayed and disturbed, but apparently it is completed, while TM healing is arrested completely [15, 20–22]. Delayed healing of TM perforations was also observed in urokinase-type plasminogen activator-deficient mice [23].

Our previous study performed on rat’s TM using Rat Wound Healing RT2 Profiler PCR Array revealed upregulated expression of mRNA for *Plat* (tissue-type plasminogen activator), *Plau* (urokinase-type plasminogen activator), *Plaur* (urokinase-type plasminogen activator receptor), and *Pai1* (plasminogen activator inhibitor type-1) in the course of healing TM perforations [24]. However, the expression of corresponding proteins in normal TM and during its healing was not examined so far.

Therefore, the aim of this study was the evaluation of protein expression creating plasminogen activation and inhibition system: urokinase-type plasminogen activator (uPA), tissue-type plasminogen activator (tPA), urokinase-type plasminogen activator receptor (uPAR), and plasminogen activator inhibitor type 1 (PAI-1) during TM healing process using Western blot method. Moreover, for the assessment of their tissue distribution, immunofluorescent method was used.

Evaluation of the involvement of plasminogen activating and inhibiting molecules in successive phases of TM healing process may create basis for the application of these molecules in the acceleration of TM healing.

## Material and Methods

### Animals, Tympanic Membrane Perforation, and Sample Collection

All animal procedures were performed in compliance with ARRIVE guidelines and the EU Directive 2010/63/EU and were approved by the Local Ethical Commission for Animal Experiments in Olsztyn (University of Warmia and Mazury, Faculty of Veterinary Medicine), Approval number: 101/2019.

Seventy wild-type Wistar rats, weighting 250–300 g, were used—male only in order to avoid the influence of the estrus cycle. Sixty animals were anaesthetized by intraperitoneal injection of 0.1 mL/100 g of equal parts of 20 mg/mL xylazine hydrochloride and 100 mg/mL ketamine hydrochloride. Before surgery ears of all rats were examined otomicroscopically to verify whether the status of the middle ear was normal. No animals were excluded from the study due to middle ear inflammatory process before or in the course of the experiment. TM perforations were performed according to the method described in our previous publications [6, 7, 24, 25]. Perforations engulfing the whole upper anterior quadrant of TM in both ears were performed under an operating microscope with a sterile, sharp point, microsurgical needle. Ten rats served as the control group, and their TM was not perforated. The rats were randomly allocated into six time-point groups (10 rats in each group) and sacrificed on days 1, 2, 3, 5, 7, and 10 after perforation by aortic exsanguinations under deep anesthesia with 100 mg/kg of pentobarbital. Before sacrification the ears were examined otomicroscopically to assess a healing process. After decapitation of 8 animals from each group, the temporal bones were removed immediately, the tympanic bulla opened under the operating microscope, the TM dissected out, immediately frozen in liquid nitrogen, and stored at − 80 °C for Western blot analysis. The TM of the remaining two animals from each group were dissected from the temporal bone together with the bony frame and fixed in 4% buffered formalin, decalcified, and processed for histological evaluation.

### Morphological Evaluation

Formalin-fixed, paraffin-embedded tissue blocks were serially sectioned into 5-µm slices and stained with hematoxylin and eosin (H&E) for basic histological assessment or used for immunofluorescence staining showing tissue distribution of proteins sought. The sections for immunofluorescence were processed and blocked as previously described [25]. The primary antibodies against tPA (bs-1545R, Bioss Antibodies, RRID: AB_10855981), uPA (FNab09267, Wuhan Fine Biotech Co.), uPAR (bs-1927R, Bioss Antibodies, RRID:AB_10856881), and PAI-1 (SERPINE1, bs-6562R, Bioss Antibodies, RRID: AB_11114126) were applied at 1:100 dilution in PBS for 90 min at room temperature. Subsequently, slides were washed and incubated with appropriate secondary antibodies conjugated with biotin (Donkey Anti-Rabbit IgG, Jackson Immuno Research Laboratories, Inc., RRID: AB_2534713) at dilution 1:200 in PBS for 1 h at room temperature, followed by washing with PBS with Tween® 20 (Sigma). Then sections were incubated with Alexa Fluor® 488, conjugated with streptavidin (S11223, Thermo Fisher) at dilution 1:1000 in PBS for 30 min in room temperature in the dark, and washed again and cell nuclei counterstained blue with Hoechst 33258 (Thermo Fisher) in PBS for 2 min at room temperature in the dark. The sections were mounted in Dako Mounting Medium on glass slides. Each staining was performed in parallel with a negative control omitting the primary antibody. Images showing tissue distribution of the immunolabeled antigens were acquired with a fluorescent microscope (Olympus BX 41 equipped with the epifluorescence module and Olympus CX30 camera) using Olympus UPlanFLN 40x/0.75 and Olympus PlanCN 20x/0.40 objectives.

### Western Blot Analysis

The quantitative evaluation of the proteins sought was performed using the Western blot method as described in our previous papers [7, 25] with slight modifications. To ensure sufficient amount of protein for the analysis, eardrums from both ears of the same animal were pooled together and homogenized on ice in RIPA buffer (Sigma) containing protease and phosphatase inhibitors (Sigma) using a manual homogenizer. The homogenates were centrifuged at 4 °C for 5 min at 8000 rpm. The supernatant was collected, and the protein concentration was measured using Bradford reagent (Bio-Rad). Samples were frozen at − 80 °C until further analysis. Thirty micrograms of protein from each sample was loaded, and slab SDS/PAGE was performed according to the method of Laemmli [26] in 8% or 12% polyacrylamide gels and blotted onto 0.2-µm pore-size nitrocellulose membranes (Bio-Rad, #1,620,112). Equal loading, electrophoresis, and transfer quality were confirmed using Ponceau Red staining (P3504, Sigma). Membranes were blocked with 5% non-fat dry milk for 1 h at room temperature. Primary antibodies recognizing rat’s tPA (PLAT, bs-1545R, Bioss Antibodies, 1:1000), uPA (PLAU, FNab09267, Wuhan Fine Biotech Co., 1:1000), uPAR (bs-1927R, Bioss Antibodies, 1:1000), and PAI-1 (SERPINE1, bs-6562R, Bioss Antibodies, 1:1000) were used. Secondary antibodies were conjugated with horseradish peroxidase (Goat Anti-Rabbit IgG, 31,462, Thermo Scientific, RRID: AB_228338, 1:15,000). Antibodies used in the study were validated and are registered in the AntibodyRegistry.org database. Blots were visualized using enhanced chemiluminescence reaction (Thermo Scientific) and exposed on X-ray film (X-Omat Blue, Carestream). Results were scanned and quantified using ImageJ. The intensity of the band of interest was related to the intensity of the whole lane stained with Ponceau S. This normalization method was validated and is being used as an alternative to actin [27].

### Statistical Analysis

Statistical analysis of protein expression evaluated in Western blot method was performed using Statistica 12. All data was first assessed for normality using the Shapiro–Wilk test and for homogeneity of group variances using the Brown-Forsythe test. In the present study, only results of tPA expression met ANOVA requirements (normal distribution and homogeneity of group variances). Therefore, results of tPA expression were evaluated by one-way analysis of variance (ANOVA) with Tukey’s post hoc test, and results of uPA, uPAR, and PaI-1 expression were evaluated using Welch’s ANOVA and post hoc Dunnett’s test. The differences were considered significant at *p* < 0.05.

## Results

### Otoscopic and Histological Observations

**Fig. 2:**
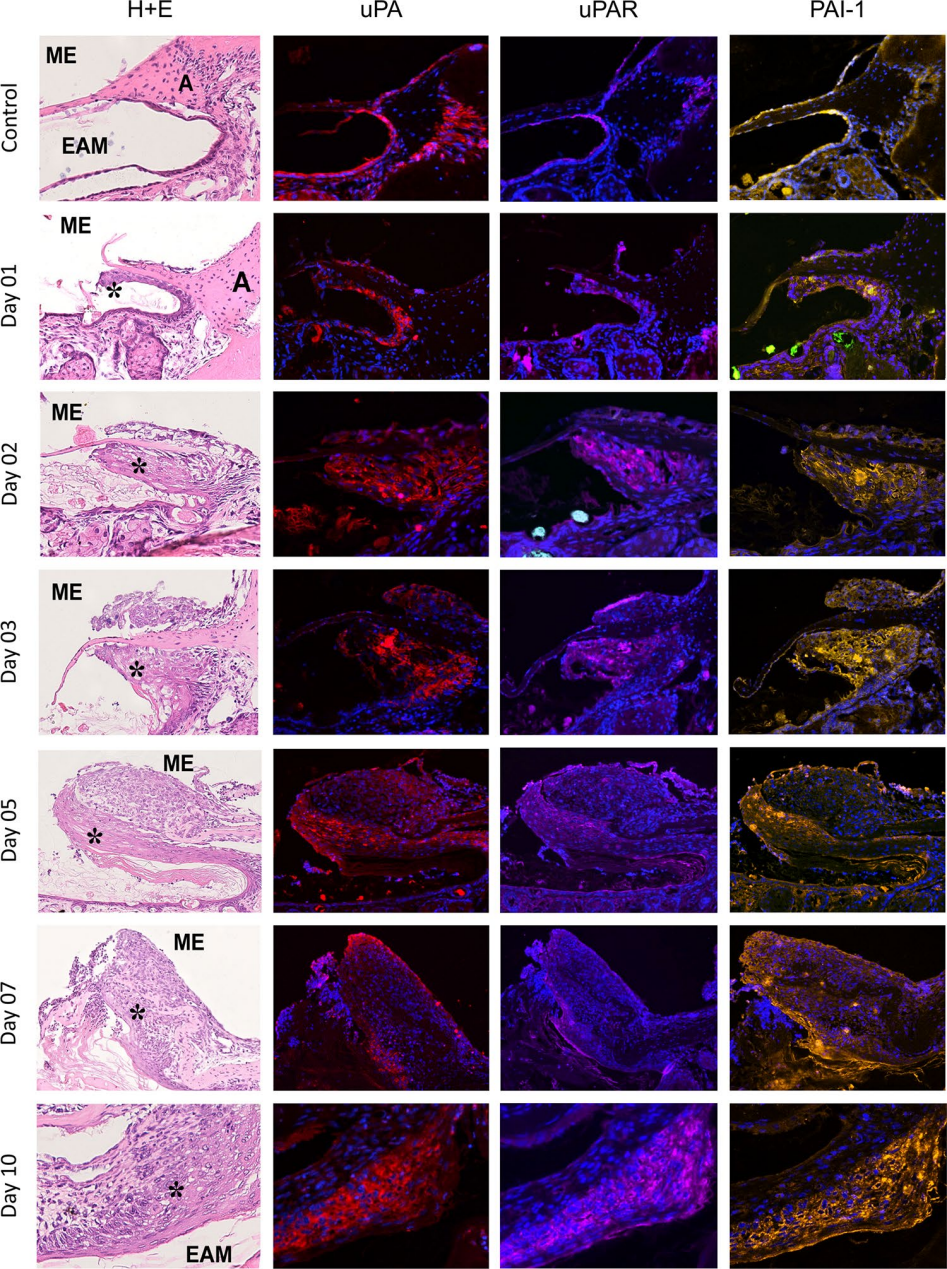
H&E and immunofluorescent staining for uPA (red), uPAR (pink), and PAI-1 (yellow), cell nuclei are stained blue. Magnification × 20 and × 40 on day 10. ME, middle ear; EAM, external acoustic meatus; A, tympanic annulus; single asterisk indicates proliferating epithelium control. *Pars tensa* of the TM, area around the annulus (H&E). Immunofluorescent staining visible in the thin layer of epithelium covering external surface of TM, mainly in the annular region, and in the epithelium covering neighboring meatus. Staining is also visible in the outer portion of annulus, especially prominent for uPA, and weak for uPAR and PAI-1. Day 1, the area near the annuls with proliferating epithelium, migrating on the surface of TM remnant (H&E). Immunofluorescent staining visible in the proliferating epithelium. The green signal visible on PAI-1 stain presumably comes from sebum autofluorescence. Day 2, the same area as on day 1 with thicker layer of proliferating epithelium migrating on TM remnant (H&E). Immunofluorescent staining visible in the proliferating epithelium. The green signal visible on PAI-1 stain presumably comes from sebum autofluorescence. Day 3, thick layer of proliferating epithelium on the external surface and thinner on the inner surface of TM in the vicinity of annulus (H&E). Immunofluorescent staining visible in the proliferating epithelium on the external surface of TM and in the superficial layer of proliferating epithelium on the inner side. Day 5, the edge of perforation and remnant of fibrous layer of *pars tensa* surrounded by proliferating squamous epithelium (H&E). Immunofluorescent staining prominent in the layer of proliferating squamous epithelium especially close to the growing front. Weak staining for uPA and uPAR is also present in proliferating fibroblasts. Day 7, the edge of perforation and remnant of fibrous layer of *pars tensa* surrounded by proliferating fibroblasts and thinner than on day 5 proliferating squamous epithelium on external side of TM. Keratin squames with inflammatory cells on its front (H&E). Immunofluorescent staining visible in the layer of proliferating squamous epithelium and in the thin mucosal layer. Day 10, closed perforation with the layer of squamous epithelium consisting of several layers and loose connective tissue composed of fibroblasts (H&E). Immunofluorescent staining clearly visible in the epithelial layer

**Fig. 3:**
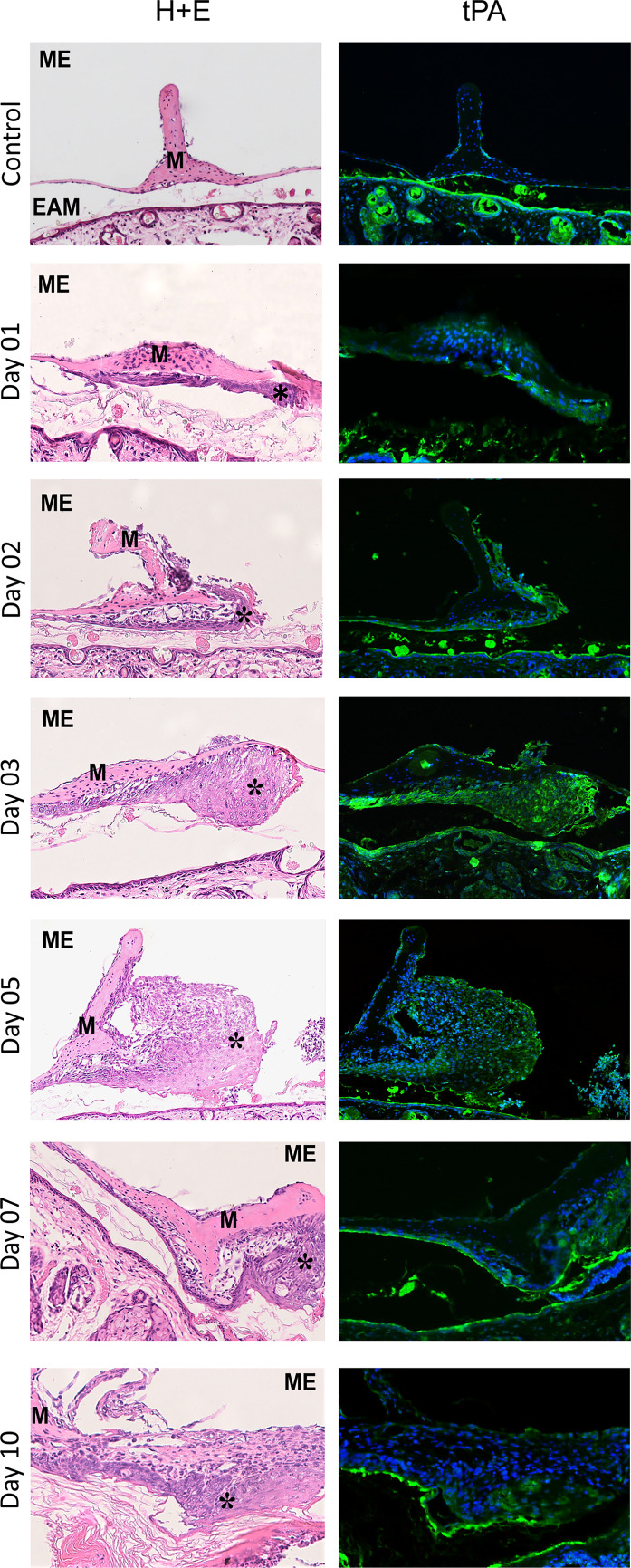
H&E and immunofluorescent staining for tPA (green), cell nuclei are stained blue. Magnification × 20 and on day 10 × 40. ME, middle ear; EAM, external acoustic meatus; M, malleus handle; single asterisk indicates proliferating epithelium control. *Pars tensa* of TM, section through malleus handle (H&E). Immunofluorescent staining for tPA is visible in the thin layer of epithelium covering external surface of the TM mainly in the malleus region and in the superficial layers of epithelium covering external acoustic meatus. The green signal visible inside the EAM space and in the sebaceous glands presumably comes from sebum autofluorescence. Day 1, the area near the umbo with a layer of proliferating epithelium (H&E). Immunofluorescent staining for tPA visible in the proliferating epithelium. Day 2, section through the malleus handle with proliferating epithelium covering the surface of malleus handle and surrounding it on the side of perforation (H&E). Immunofluorescent staining for tPA visible in the proliferating epithelium. Day 3, section through the lower part of the malleus handle, the ridge of proliferating epithelial cells near the malleus, migrating on the external surface of TM remnant (H&E). Immunofluorescent staining for tPA visible in the proliferating epithelium. Day 5, section through the malleus handle with proliferating epithelium and thick layer of proliferating fibroblasts adjacent to the perforation (H&E). Immunofluorescent staining for tPA clearly visible in the layer of proliferating squamous epithelium. Day 7, section through the malleus handle with proliferating epithelium (H&E). Immunofluorescent staining for tPA visible in the proliferating epithelium. Day 10, closed perforation with uneven layer of squamous epithelium and loose connective tissue composed of fibroblasts (H&E). Immunofluorescent staining for tPA visible in the epithelium, especially in its superficial layer

Otoscopic and histological observations corresponding to the one reported in our previous publications [7, 25] were summarized in Table 1. Histological examinations were performed on horizontal sections through the perforations localized in the anterior part of TM *pars tensa* as presented on Fig. 1A. Histological pictures of TM perforations healing process in consecutive days after surgery stained with H&E were presented in the first column of Fig. 2 (area adjacent to the tympanic annulus) and of Fig. 3 (area adjacent to the malleus handle).

**Table 1:** Otoscopic and histological observations of TM healing process

Day	Otoscopic observations (10 animals—20 ears from each day)	Histological examinations (3 animals—6 ears from each day)
01	Opaque circle in some distance from the edges of perforation, size of the perforations unchanged	Near annulus and malleus handle small area of proliferating epithelium
02	Opaque circle in some distance from the edges of perforation, size of the perforations unchanged	Near annulus and malleus handle greater area of proliferating epithelium
03	Perforations reduced by about 1/3 with markedly thickened edges	Thick layer of proliferating epithelium progressing from annulus and malleus handle area towards perforation
05	Perforations further reduced up to ½ with thickened edges covered by crust	Perforations partially covered by thick layer of squamous epithelium and fibrous layer rich in fibroblasts underneath
07	Perforations almost closed, surrounded by the crust with small hole in the middle	TM around the persisting perforations thickened, consisting of thick squamous epithelium and a loose fibrous layer
10	All but 3 ears perforations closed by thick, whitish scar sometimes covered with crust. In 3 ears, perforations were very small but still opened	Perforations were closed by the thick layers of squamous epithelium and fibroblasts. In one ear, the center of perforation was not closed, still filled with keratin squames and amorphous substance with granulocytes

### Immunofluorescence Assessment

The assessment of tissue localization of uPA, uPAR, tPA, and PAI-1 with immunofluorescent staining in control animals revealed reaction in the thin layer of epithelium covering external surface of the TM mainly in the annular region, on the corner between TM and external acoustic meatus (EAM) skin, as well as in the area of the malleus handle. The expression was also visible in the superficial layers of epithelium covering EAM near the TM. In the outer, more cellular, and vascular portion of fibrous annulus, clear immunofluorescent reaction was particularly pronounced for uPA and tPA (Figs. 2 and 3).

In TM with healing perforations, tissue distribution of all examined elements of plasminogen activation and inhibition system was similar. Their presence was detected in the proliferating and migrating epithelium on days 1 to 3 close to the annulus and malleus handle and on subsequent days in the epithelium surrounding the perforation, overhanging its border, and gradually covering it. In the layer composed of fibroblasts, immunofluorescent reaction for all tested molecules was very weak (Figs. 2 and 3).

### Western Blot Analysis

**Fig. 4:**
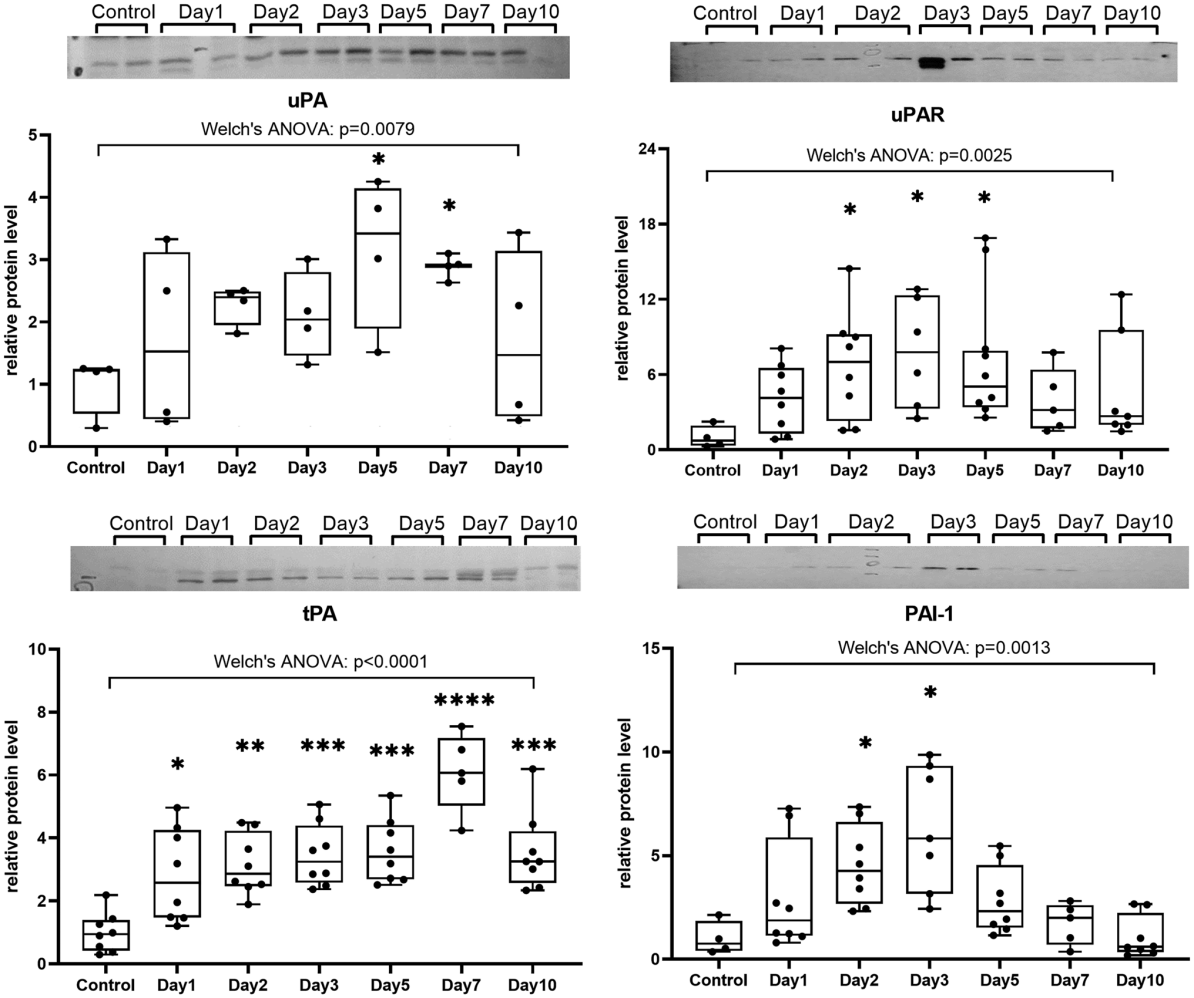
Expression of uPA, uPAR, tPA, and PAI-1 proteins in consecutive time points after TM perforation evaluated using Western blot technique. Individual values are marked with dots, boxes represent 25–75 percentile; the line inside the box—the median and the whiskers indicate the minimal and maximal values obtained from 4 to 8 animals in each time point. The abundance of the protein was related to its expression in the control group, which was set as 1. *p < 0.05; **p < 0.01; ***p < 0.001; ****p < 0.0001 (ANOVA and post hoc Tukey’s test or Welch’s ANOVA and post hoc Dunnett’s test)

Western blot analysis was performed to determine the evolution of plasminogen activation system elements (uPA, uPAR, tPA, and PAI-1) in the course of TM healing. The quantitative densitometry data is presented in Fig. 4. Whole Western blots are presented in Suppl. Fig. S1. The level of uPA expression showed upregulation, starting soon after injury and reached the level of statistical significance on days 5 and 7 (*p* < 0.05), and then it decreased on day 10. Welch’s ANOVA of six experimental groups, presenting results obtained in six consecutive time points of TM healing, and control group yielded *W*(6, 8.77) = 6.35, *p* = 0.0079. Further post hoc comparisons between control and experimental groups with Dunnett’s test revealed statistically significant increase of uPA expression on days 5 and 7 (*p* < 0.05).

The elevation uPAR expression also started from day 1 and lasted until the end of observation period with the highest expression on days 2, 3, and 5. Welch’s ANOVA of six experimental groups and control group yielded *W*(6, 16.59) = 5.56, *p* = 0.0025. Post hoc comparisons performed with Dunnett’s test showed that statistically significant raise of uPA took place on days 2, 3, and 5 (*p* < 0.05) as compared to the control group.

The tPA expression showed abrupt increase and was about three times higher on the first day after injury than in control group and maintained that level up to day 5. The maximum expression level was observed on day 7. On day 10, the level of expression returned to the ones observed in the days 3 and 5. ANOVA of six experimental groups and control group yielded *F*(6, 49) = 11.64, *p* < 0.0001. Post hoc comparisons with Tukey’s test revealed statistically significant differences throughout the whole period of observation in comparison to the control group (day 1 *p* < 0.05; day 2 *p* < 0.01; day 3, day 5, and day 10 *p* < 0.001; and day 7 *p* < 0.0001).

PAI-1 showed steady increase in the expression starting soon after injury with the highest expression on day 3, followed by gradual attenuation. Welch’s ANOVA of six experimental groups and control group yielded *W*(6, 25.98) = 6.28, *p* = 0.0013. Post hoc comparisons performed with Dunnett’s test revealed statistically significant differences on day 2 and day 3 (*p* < 0.05) compared to the control group.

## Discussion

Plasminogen activation system is regulated by the balance between plasminogen activators and inhibitors [28]. There are two major types of plasminogen activators: tPA, which provides efficient cell-independent fibrinolysis due to its high affinity to fibrin, and uPA, which mediates cell surface proteolysis and plasminogen activation important for resolution of fibrin-rich wound fields in the skin and for keratinocytes migration [29].

The uPA activity is directed to cellular surfaces through its high-affinity receptor uPAR. The uPAR mediates activation of intracellular signaling cascades including the tyrosine kinase Src, the serine kinase Raf, focal adhesion kinase (FAK), extracellular-signal-regulated kinase (ERK)/mitogen-activated protein kinase (MAPK) important for cell adhesion, migration and growth in keratinocytes, fibroblasts, neutrophils, and endothelial cells [28, 30]. High expression of this receptor was observed in organs undergoing extensive tissue remodeling, for example, migrating keratinocytes at the edge of skin wounds [31].

PAI-1 is the primary inhibitor of both tPA and uPA. Its binding to vitronectin enables the stabilization and control of proteolytic events. Plasmin, through activation of TGF β, stimulates the expression of PAI-1 and simultaneously suppresses the expression of uPA which makes up self-control feedback mechanism within plasminogen activation and inhibition system [28]. Although PAI-1 is a trace plasma protein, it is present in relatively high concentration in platelets, and it stabilizes newly formed thrombi [32]. Moreover, PAI-1 plays a leading role in migration of cells by regulating ECM proteolysis and by detachment of cells’ surface integrins from their ECM ligands [33, 34]. Therefore, PAI-1 should be considered a multipurpose protease inhibitor, which participates in all stages of tissue repair, regulation of coagulation, fibrinolysis, inflammation, cell migration, and stromal remodeling [35].

The role of the plasminogen activation system in wound healing was first discussed in terms of the degradation of fibrin necessary for clot resolution. Its influence on ECM remodeling and cell migration, including keratinocyte migration over the wound area has been recognized much later. Nowadays, several other functions have been assigned to this system: regulation of the inflammatory phase, activation of growth factors, promotion of angiogenesis, and granulation tissue formation [15–17, 30, 36].

The studies based on knock-out mice documented a vital importance of the plasminogen system for skin wound healing. In plg-deficient as well as tPA/uPA double-deficient mice, the healing of cutaneous wounds was delayed, but complete wound closure was ultimately achieved. The wound closure and epidermal re-epithelialization were significantly less impaired in uPA/tPA double-deficient mice than in plasminogen-deficient mice [29, 37–39].

The similar studies concerning TM healing showed that in plasminogen-deficient mice, healing was completely arrested [20, 22]. In these animals, the recruitment of the inflammatory cells to the wound area was similar, but tissue debridement, removal of fibrin, resolution of inflammatory reaction, keratinocyte migration, and in-growth of connective tissue were impaired. In wild-type mice, all perforations were closed within 11 days, while in plasminogen-deficient mice, five of 26 TM perforations were still open after 50 days. Even when the perforations seemed to be otomicroscopically closed, morphological studies revealed that they filled up with amorphous mass of fibrin and inflammatory cells. Although the proliferation of the keratinocytes was observed in plasminogen-deficient mice, they appeared to be detached from each other, and their migration towards the center of the perforation was significantly inhibited.

Similar abnormalities in histological appearance were also observed in uPA-knock-out mice, although TM healing was much less impaired, and all perforations were finally closed on day 15 in otomicroscopic and in immunohistochemical evaluations [23]. It is however worth to note that plasminogen or uPA-deficient mice in the course of life spontaneously develop chronic otitis media with extensive fibrin deposition in middle ear cavity, which may also contribute to delay of TM healing [21, 23]. The crucial role of plasminogen in TM healing was confirmed by studies showing that plasminogen injection restored TM healing ability in plasminogen-deficient mice and accelerated the healing rate in wild-type mice [20, 40]. Although mentioned studies evidenced the significance of plasminogen system in TM healing, the expression of molecules creating this system in various phases of healing process was not examined so far.

Our previous study performed on rats’ perforated TM in successive time points of healing process using Rat Wound Healing RT2 Profiler PCR Array showed upregulation of Plau, Plat, Plaur, and PaI-1 genes [24]. Western blot analysis performed in this study revealed elevated expression of uPA during inflammatory and early proliferative phases of TM healing. Statistically significant upregulation was observed on days 5 and 7, when proliferation of keratinocytes and fibroblasts was most intensive, with subsequent drop on day 10 during remodeling phase. The uPAR expression exhibited similar to uPA upregulation starting from day 1 with the highest values on days 2, 3, and 5, when the keratinocyte migration was especially active. PAI-1 expression showed steady increase achieving the highest level on day 3 with subsequent gradual weakening. Its statistically significant increase was observed on days 2, 3, and 5. Our results are consistent with other observations that uPA and PAI-1 are among highly and early induced genes that typify the migratory epithelium [41]. The moving cells need both uPA (via interaction with uPAR) and PAI-1 in the leading edge which explains their simultaneous expression [33, 35]. PAI-1 is also synthesized in response to many different factors like TGFβ, released by inflammatory cells, and thus it may be upregulated independently from tPA/uPA-plasmin pathway [35].

Immunofluorescence evaluation revealed the expression of all components of the plasminogen activation and inhibition system in the proliferating and migrating epithelium of the healing TM throughout the observation period. These results are in line with the results of the studies of skin wound healing [31, 42–44].

In the skin wounds of mice, uPA immunoreactivity was confirmed in virtually all keratinocytes at the edge of the wound after 12 h and in days 2–10 after injury. In human skin wounds, immunoreactivity of uPA was observed in 2nd and 5th day after wounding also in keratinocytes of epithelial outgrowths. Mouse skin wounds showed also strong uPAR reactivity in the migrating but not resting keratinocytes [31].

Rømer et al. [42, 43] examined expression of uPA, uPAR, and PAI-1 mRNA in the mouse skin wounds using in situ hybridization. The expression of mRNA for these molecules was present in keratinocytes of the epithelial outgrowths. In the immunohistochemical studies, uPA protein was detected in keratinocytes in several layers of the epithelial outgrowth, whereas PAI-1 protein was limited to the basal keratinocytes adjacent to the area of the basal membrane.

In Western blot analysis presented in the current study, high expression of tPA was observed in all phases of TM healing, with the highest activity during remodeling phase. Immunofluorescent staining showed its expression in the migrating epithelium. The tPA focuses plasminogen activation on fibrin surfaces which is important in degradation of the fibrin clot on the edge of TM perforation [16, 44, 45], although studies on tPA-deficient mice did not show delayed closing of TM perforations [23]. Also, the studies on mice deficient of various plasminogen activation system elements showed that the uPA alone sufficiently supplies fibrinolytic potential to clear fibrin deposits and support wound healing without the benefit of the tPA that suggests functional overlap between these two molecules [29]. Nevertheless, studies of skin wounds, likewise of TM perforations healing, showed that tPA undoubtedly plays a role in this process [44]. The presence of tPA mRNA was detected in human skin wounds and ulcers in the basal epidermis at the ulcer margin, and tPA protein in all layers of epithelium, while in mouse wounds, tPA immunoreactivity was observed from days 5 to 10 in keratinocytes located superficially in the epidermal outgrowths [46]. Having said that, scant literature concerning the role of tPA in wound healing makes the interpretation of its relationship with other plasminogen system molecules difficult.

Contrary to observations in the normal skin epidermis, where expression of uPA, uPAR, and PAI-1 was not found using immunohistochemical and in situ hybridization methods [31, 42, 43, 46], in our immunofluorescence evaluation, the presence of these molecules was confirmed in the epithelium forming migratory centers of normal TM and in the epithelium covering EAM. These differences may result from unique features of TM and EAM epithelium and its peculiar migratory capacity. Keratinocytes covering mentioned surfaces express cytokeratin typical for proliferating and migrating epithelium not observed in normal epidermis. In TM keratinocyte migration occurs in radial and centrifugal and on bony parts of external acoustic meatus in lateral direction [9, 47]. This mechanism ensures the self-cleansing function of TM and acoustic meatus, preventing accumulation of desquamation products. Expression of plasminogen activating and inhibiting molecules in normal epithelium of these areas suggests that they are required to maintain the migratory function of this epithelium and for debris removal. This presumption is further supported by the observation that in plasminogen- and uPA-deficient mice, keratin formation in the EAM is impaired.

Enhanced expression of tPA and uPA proteins during TM healing indicates that both plasminogen activators are involved. Upregulation of uPA and PAI-1 during the proliferation phase of TM healing reflects their involvement mainly in epithelial cell migration, while the highest expression of tPA in the final phase of healing suggests its role in termination of TM healing and formation of the scar.

Explanation of the role played by plasminogen activating and inhibiting molecules in particular phases of TM healing process may constitute the basis for development of future treatment strategies.

### Limitations of the Study

Our study was performed only on male rate; thus, interpolation of these results on female may be limited.

The control animals used in this study were not sham-operated and were not subjected to associated anesthesia. It may be considered a limitation of the study; however, the perforation of TM was performed under short general anesthesia to avoid any procedure-related pain or discomfort. In our opinion, this should not affect protein expression in TM.

## Supplementary Information

Below is the link to the electronic supplementary material.

Supplementary file1 (TIF 2678 kb)

## Data Availability

The raw data of the study are available in Department of General and Experimental Pathology, Medical University of Białystok, Mickiewicza 2c, 15-222 Białystok, Poland, Magdalena Cieślińska, e-mail: magdalena.cieslinska@umb.edu.pl

## References

[1] Reijnen C, Kuijpers W (1971). The healing pattern of the drum membrane. Acta Otolaryngol Suppl 287:1–74.Blasi F, Sidenius N. (2010) The urokinase receptor: Focused cell surface proteolysis, cell adhesion and signaling. FEBS Lett.

[2] Stenfors L-E, Carlsöö B, Salén B, Winblad B (1980). Repair of experimental tympanic membrane perforations. Acta Otolaryngol.

[3] Johnson AP, Smallman LA, Kent SE (1990). The mechanism of healing of tympanic membrane perforations. Acta Otolaryngol.

[4] Wang WQ, Wang ZM, Chi FL (2004). Spontaneous healing of various tympanic membrane perforations in the rat. Acta Otolaryngol.

[5] Santa Maria PL, Redmond SL, Atlas MD, Ghassemifar R (2010). Histology of the healing tympanic membrane following perforation in rats. Laryngoscope.

[6] Zajączkiewicz H, Hassmann-Poznanska E, Skotnicka B, Chyczewski L, Reszeć J, Winnicka MM (2014). The healing process of tympanic membrane perforations in rats. Otolaryngol Pol.

[7] Makuszewska M, Sokołowska M, Hassmann-Poznańska E, Bialuk I, Skotnicka B, Bonda T, Reszeć J, Winnicka M (2015). Enhanced expression of hepatocyte growth factor in the healing of experimental acute tympanic membrane perforation. Int J Pediatr Otorhinolaryngol.

[8] Knutsson J, von Unge M, Rask-Andersen H (2011). Localization of progenitor/stem cells in the human tympanic membrane. Audiol Neurotol.

[9] Kim SW, Kim J, Seonwoo H, Jang K, Kim Y, Lim H, Lim K, Tian C, Chung J, Choung Y (2015). Latent progenitor cells as potential regulators for tympanic membrane regeneration. Sci Rep.

[10] Jo M, Lester RD, Montel V, Eastman B, Takimoto S, Gonias SL (2009). Reversibility of epithelial-mesenchymal transition (EMT) induced in breast cancer cells by activation of urokinase receptor-dependent cell signaling. J Biol Chem.

[11] Omori K, Hattori N, Senoo T, Takayama Y, Masuda T, Nakashima T, Iwamoto H, Fujitaka K, Hamada H, Kohno N (2016). Inhibition of plasminogen activator inhibitor-1 attenuates transforming growth factor-β-dependent epithelial mesenchymal transition and differentiation of fibroblasts to myofibroblasts. PloS one.

[12] Yamagami Y, Kawami M, Ojima T, Futatsugi S, Yumoto R, Takano M (2020). Role of plasminogen activator inhibitor-1 in methotrexate-induced epithelial-mesenchymal transition in alveolar epithelial A549 cells. Biochem Biophys Res Commun.

[13] Santa Maria PL, Kim S, Varsak YK, Yang YP (2015). Heparin binding-epidermal growth factor-like growth factor for the regeneration of chronic tympanic membrane perforations in mice. Tissue Eng - Part A.

[14] Santa Maria PL, Santa Maria C, Kim S, Yang YP (2016). Single administration of a sustained-release formulation of KB-R7785 inhibits tympanic membrane regeneration in an animal model. J Int Adv Otol.

[15] Sulniute R, Shen Y, Guo YZ, Fallah M, Ahlskog N, Ny L, Rakhimova O, Broden J, Boija H, Moghaddam A, Li J, Wilczynska M, Ny T (2016). Plasminogen is a critical regulator of cutaneous wound healing. Thromb Haemost.

[16] Heissig B, Salama Y, Takahashi S, Osada T, Hattori K (2020). The multifaceted role of plasminogen in inflammation. Cell Signal.

[17] Shen Y, Guo Y, Mikus P, Sulniute R, Wilczynska M, Ny T, Li J (2012). Plasminogen is a key proinflammatory regulator that accelerates the healing of acute and diabetic wounds. Blood.

[18] Ny L, Parmer RJ, Shen Y, Holmberg S, Baik N, Bäckman A, Broden J, Wilczynska M, Ny T, Miles L (2020). The plasminogen receptor, Plg-RKT, plays a role in inflammation and fibrinolysis during cutaneous wound healing in mice. Cell Death Dis.

[19] Voegeli R, Rawlings AV, Haftek M (2019). Expression and ultrastructural localization of plasmin(ogen) in the terminally differentiated layers of normal human epidermis. Int J Cosmet Sci.

[20] Li J, Eriksson PO, Hannson A, Hellström S, Ny T (2006). Plasmin/plasminogen is essential for the healing of tympanic membrane perforations. Thromb Haemost.

[21] Eriksson PO, Li J, Ny T, Hellström S (2006). Spontaneous development of otitis media in plasminogen-deficient mice. Int J Med Microbiol.

[22] Prestwich A, Li J, Eriksson PO, Ny T, Berggren D, Hellström S (2008). Lack of plasminogen does not alter the early inflammatory response following a tympanic membrane perforation: a study in plasminogen-deficient mice. Acta Otolaryngol.

[23] Shen Y, Guo Y, Du C, Wilczynska M, Hellström S, Ny T (2012) Mice deficient in urokinase-type plasminogen activator have delayed healing of tympanic membrane Perforations. PLoS One 7. 10.1371/journal.pone.0051303 10.1371/journal.pone.0051303 PMC3517469 23236466

[24] Hassmann-Poznańska E, Taranta A, Bialuk I, Poznańska M, Zajaczkiewicz H, Winnicka MM (2013). Analysis of gene expression profiles in tympanic membrane following perforation using PCR Array in rats-Preliminary investigation. Int J Pediatr Otorhinolaryngol.

[25] Makuszewska M, Bonda T, Cieślińska M, Bialuk I, Winnicka M, Skotnicka B, Hassmann-Poznańska E (2019). Expression of collagens type I and V in healing rat’s tympanic membrane. Int J Pediatr Otorhinolaryngol.

[26] Laemmli UK (1970). Cleavage of structural proteins during the assembly of the head of bacteriophage T4. Nature.

[27] Romero-Calvo I, Ocón B, Martínez-Moya P, Suárez MD, Zarzuelo A, Martínez-Augustin O, Sánchez de Medina F (2010). Reversible Ponceau staining as a loading control alternative to actin in Western blots. Anal Biochem.

[28] Li WY, Chong SSN, Huang EY, Tuan TL (2003). Plasminogen activator/plasmin system: a major player in wound healing?. Wound Repair Regen.

[29] Bugge TH, Flick MJ, Danton MJS, Daugherty C, Rømer J, Danø K, Carmeliet P, Collen D, Degen J (1996). Urokinase-type plasminogen activator is effective in fibrin clearance in the absence of its receptor or tissue-type plasminogen activator. Proc Natl Acad Sci USA.

[30] Blasi F, Sidenius N (2010). The urokinase receptor: focused cell surface proteolysis, cell adhesion and signaling. FEBS Lett.

[31] Solberg H, Ploug M, Høyer-Hansen G, Nielsen BS, Lund LR (2001). The murine receptor for urokinase-type plasminogen activator is primarily expressed in tissues actively undergoing remodeling. J Histochem Cytochem.

[32] Czekay RP, Loskutoff DJ (2004). Unexpected role of plasminogen activator inhibitor 1 in cell adhesion and detachment. Exp Biol Med (Maywood).

[33] Czekay RP, Wilkins-Port CE, Higgins SP, Freytag J, Overstreet JM, Klein RM, Higgins CE, Samarakoon R, Higgins PJ (2011). PAI-1: an integrator of cell signaling and migration. Int J Cell Biol.

[34] Providence KM, Higgins SP, Mullen A, Battista A, Samarakoon R, Higgins CE, Wilkins-Port CE, Higgins PJ (2008). SERPINE1 (PAI-1) is deposited into keratinocyte migration “trails” and required for optimal monolayer wound repair. Arch Dermatol Res.

[35] Simone TM, Higgins CE, Czekay RP, Law BK, Higgins SP, Archambeault J, Kutz SM, Higgins PJ (2014). SERPINE1: a molecular switch in the proliferation-migration dichotomy in wound-“activated” keratinocytes. Adv Wound Care (New Rochelle).

[36] Lund LR, Rømer J, Bugge TH, Nielsen B, Frandsen T, Degen J, Stephens R, Danø K (1999). Functional overlap between two classes of matrix-degrading proteases in wound healing. EMBO J.

[37] Rømer J, Bugge T, Pyke C, Lund L, Flick M, Degen J, Danø K (1996). Impaired wound healing in mice with a disrupted plasminogen gene. Nat Med.

[38] Lund LR, Green KA, Stoop AA, Ploug M, Almholt K, Lilla J, Nielsen B, Christensen I, Craik C, Werb Z, Danø K, Rømer J (2006). Plasminogen activation independent of uPA and tPA maintains wound healing in gene-deficient mice. EMBO J.

[39] Jögi A, Rønø B, Lund IK, Nielsen B, Ploug M, Høyer-Hansen G, Rømer J, Lund L (2010). Neutralisation of uPA with a monoclonal antibody reduces plasmin formation and delays skin wound healing in tPA-deficient mice. PLoS ONE.

[40] Shen Y, Guo Y, Wilczynska M, Li J, Hellström S, Ny T (2014). Plasminogen initiates and potentiates the healing of acute and chronic tympanic membrane perforations in mice. J Transl Med.

[41] Freytag J, Wilkins-Port CE, Higgins CE, Higgins SP, Samarakoon R, Higgins PJ (2010). PAI-1 mediates the TGF-beta1+EGF-induced “scatter” response in transformed human keratinocytes. J Invest Dermatol.

[42] Rømer J, Lund L, Eriksen J, Ralfkiaer E, Zeheb R, Gelehrter T, Dano K, Kristensen P (1991). Differential expression of urokinase-type plasminogen activator and its type-1 inhibitor during healing of mouse skin wounds. J Invest Dermatol.

[43] Rømer J, Lund LR, Eriksen J, Pyke C, Kristensen P, Danø K (1994). The receptor for urokinase-type plasminogen activator is expressed by keratinocytes at the leading edge during re-epithelialization of mouse skin wounds. J Invest Dermatol.

[44] Weckroth M, Vaheri A, Virolainen S, Saarialho-Kere U, Jahkola T, Sirén V (2004). Epithelial tissue-type plasminogen activator expression, unlike that of urokinase, its receptor, and plasminogen activator inhibitor-1, is increased in chronic venous ulcers. Br J Dermatol.

[45] Collen D, Lijnen HR (2004). Tissue-type plasminogen activator: a historical perspective and personal account. J Thromb Haemost.

[46] Grøndahl-Hansen J, Lund LR, Ralfkiær E, Ottevanger V, Dano K (1988). Urokinase- and tissue-type plasminogen activators in keratinocytes during wound reepithelialization in vivo. J Invest Dermatol.

[47] Vennix PPCA, Kuijpers W, Peters TA, Tonnaer ELGM, Ramaekers FCS (1996). Epidermal differentiation in the human external auditory meatus. Laryngoscope.

