# Supplementary figures and images for: Enhanced Expression of Plasminogen Activators and Inhibitor in the Healing of Tympanic Membrane Perforation in Rats
Source: J Assoc Res Otolaryngol. 2023 Feb 21;24(2):159–70. doi: 10.1007/s10162-023-00891-5 (PMC10121974; doi:10.1007/s10162-023-00891-5)

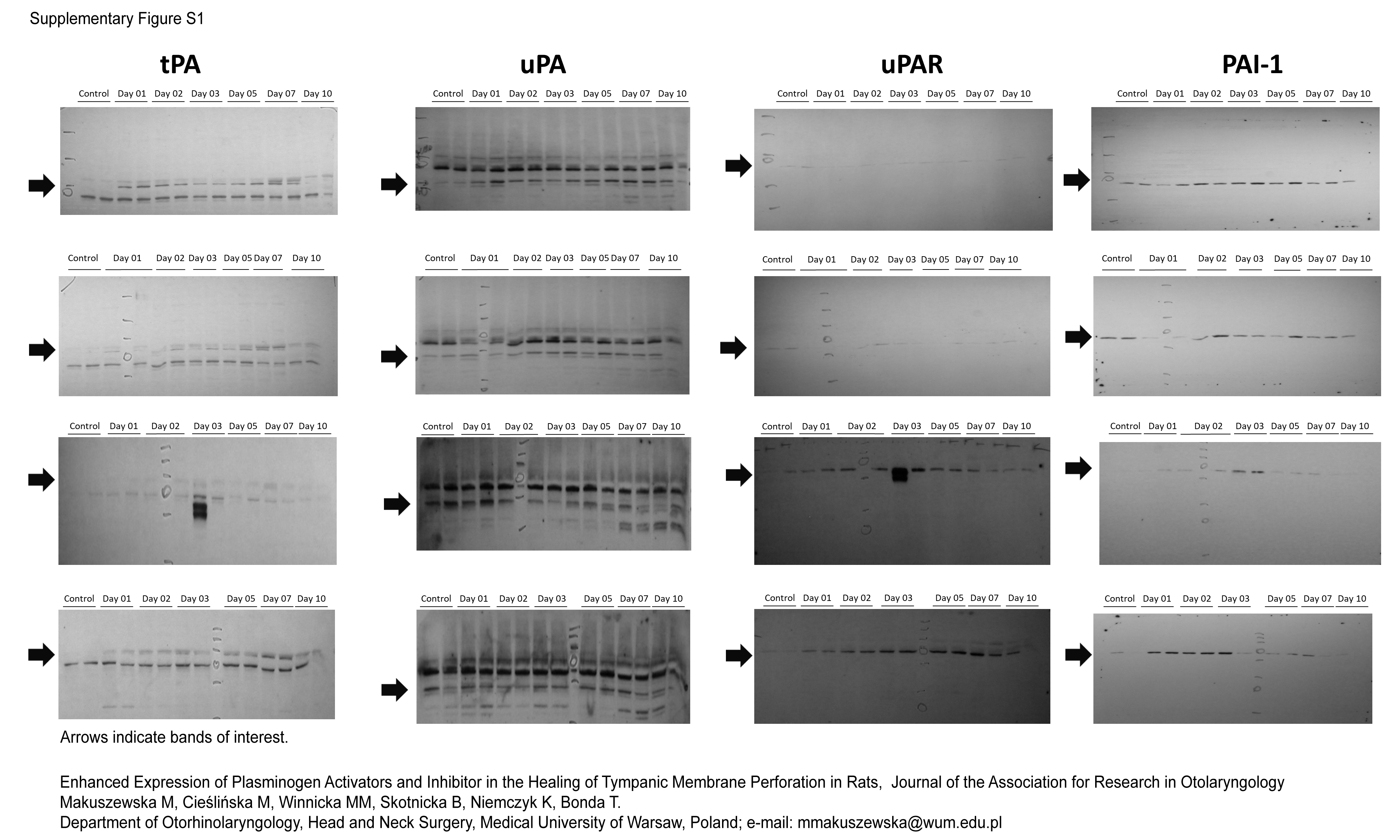

Supplement: Supplementary file 1 — Supplementary file1 (TIF 2678 kb) [file 10162_2023_891_MOESM1_ESM.tif]
